# Humans Lack iGb3 Due to the Absence of Functional iGb3-Synthase: Implications for NKT Cell Development and Transplantation

**DOI:** 10.1371/journal.pbio.0060172

**Published:** 2008-07-15

**Authors:** Dale Christiansen, Julie Milland, Effie Mouhtouris, Hilary Vaughan, Daniel G Pellicci, Malcolm J McConville, Dale I Godfrey, Mauro S Sandrin

**Affiliations:** 1 Department of Surgery, The University of Melbourne, Austin Health/Northern Health, Heidelberg, Victoria, Australia; 2 Department of Microbiology and Immunology, The University of Melbourne, Parkville, Victoria, Australia; 3 Department of Biochemistry and Molecular Biology, The University of Melbourne, Bio21 Molecular Science and Biotechnology Institute, Melbourne, Victoria, Australia; Massachusetts Institute of Technology, United States of America

## Abstract

The glycosphingolipid isoglobotrihexosylceramide, or isogloboside 3 (iGb3), is believed to be critical for natural killer T (NKT) cell development and self-recognition in mice and humans. Furthermore, iGb3 may represent an important obstacle in xenotransplantation, in which this lipid represents the only other form of the major xenoepitope Galα(1,3)Gal. The role of iGb3 in NKT cell development is controversial, particularly with one study that suggested that NKT cell development is normal in mice that were rendered deficient for the enzyme iGb3 synthase (iGb3S). We demonstrate that spliced iGb3S mRNA was not detected after extensive analysis of human tissues, and furthermore, the iGb3S gene contains several mutations that render this product nonfunctional. We directly tested the potential functional activity of human iGb3S by expressing chimeric molecules containing the catalytic domain of human iGb3S. These hybrid molecules were unable to synthesize iGb3, due to at least one amino acid substitution. We also demonstrate that purified normal human anti-Gal immunoglobulin G can bind iGb3 lipid and mediate complement lysis of transfected human cells expressing iGb3. Collectively, our data suggest that iGb3S is not expressed in humans, and even if it were expressed, this enzyme would be inactive. Consequently, iGb3 is unlikely to represent a primary natural ligand for NKT cells in humans. Furthermore, the absence of iGb3 in humans implies that it is another source of foreign Galα(1,3)Gal xenoantigen, with obvious significance in the field of xenotransplantation.

## Author Summary

Identification of endogenous antigens that regulate natural killer T (NKT) cell development and function is a major goal in immunology. Originally the glycosphingolipid, iGb3, was suggested to be the main endogenous ligand in both mice and humans. However, recent studies have challenged this hypothesis. From a xenotransplantation (animal to human transplants) perspective, iGb3 expression is also important as it represents another form of the major xenoantigen Galα(1,3)Gal. In this study, we assessed whether humans expressed a functional iGb3 synthase (iGb3S), the enzyme responsible for lipid synthesis. We showed that spliced iGb3S mRNA was not detected in any human tissue analysed. Furthermore, chimeric molecules composed of the catalytic domain of human iGb3S were unable to synthesize iGb3 lipid, due to at least one amino acid substitution. We also demonstrated that purified human anti-Gal antibodies bound iGb3 lipid and mediated destruction of cells transfected to express iGb3. A nonfunctional iGb3S in humans has two major consequences: (1) iGb3 is unlikely to be a natural human NKT ligand and (2) natural human anti-Gal antibodies in human serum could react with iGb3 on the surface of organs from pigs, marking these tissues for immunological destruction.

## Abstract 3

Controversy surrounds the glycolipid iGb3. Our data show that humans do not express this lipid. This has important implications in natural killer T cell development, self-recognition, and transplantation.

## Introduction

Identification of endogenous antigens that regulate NKT cell development and self-recognition represents a major goal in immunology. This unique population of T cells is characterised by expression of an invariant Vα14Jα18 TCR—Vα24Jα18 in humans—and the recognition of glycolipid antigens presented by CD1d [1]. When activated, natural killer T (NKT) cells regulate immune responses through their ability to produce large amounts of cytokines such as interferon (IFN)-γ and interleukin (IL)-4 [2]. NKT cell deficiencies are associated with a range of diseases, including cancer, autoimmunity, and infection, in mice and humans [2]. This, combined with the fact that NKT cell numbers vary widely in humans [3], highlights the importance of understanding the endogenous antigens in humans that regulate NKT cell development and function.

Initial work demonstrated that α-galactosylceramide (α-GalCer), a glycosphingolipid originally derived from a marine sponge [4], was a potent agonist for NKT cells in a CD1d-dependent manner in both mice and humans [5,6]. However, the physiological relevance of this in mammalian systems was difficult to understand because α-GalCer is not a mammalian product. Zhou et al. [7] demonstrated that a deficiency in the lysosomal enzymes β-hexaminidase A and B selectively abrogated NKT cell development, suggesting that glycolipid(s) downstream of these enzymes are responsible for NKT cell selection. Experiments to directly test which of the candidate glycolipids were capable of stimulating NKT cells pointed to the glycosphingolipid, isogloboside 3 (iGb3). Both mouse and human fresh NKT cells, and NKT cell hybridomas and lines, responded to iGb3, and furthermore, specific inhibition of iGb3 on human cells, using isolectin B4 (IB4) that should selectively target iGb3 via its terminal Galα(1,3)Gal sugars, suggested that iGb3 was also a primary human self-antigen for NKT cells [7]. These data led the authors to suggest that iGb3 was the main endogenous ligand responsible for NKT cell development and self-recognition in both mice and humans. Subsequent studies from independent groups have confirmed that iGb3 is an agonist ligand for at least a subset of mouse and human NKT cells [8–13], and furthermore, that this glycosphingolipid appears to be important for shaping the NKT cell TCR repertoire in mice [12]. However, recent studies have challenged the hypothesis that iGb3 is the primary ligand responsible for NKT cell development in mice [14–16]. One of these studies [16] failed to detect iGb3 in mouse or human thymus, although this study could not exclude the existence of low levels of iGb3, or higher levels of iGb3 expressed by a minor subset of the thymus. Another study [15] demonstrated, by using iGb3 synthase (iGb3S) knockout mice, that NKT cell development was apparently normal, which more strongly suggested that iGb3 is at least not essential for this process in mice. Lastly, two papers have provided evidence that the defect in NKT cell development in Hex-b–deficient mice may be the lysosomal storage disease that occurs with this mutation, thus causing a nonspecific defect in glycolipid processing and presentation [14,17,18]. The ability of iGb3 to activate human NKT cells is not in dispute; what remains in question is the role of iGb3 in human NKT cell biology.

Another issue of major importance involving iGb3 is from the perspective of xenotransplantation, in which expression of this glycolipid on the cell surface of pig tissue could represent a major problem if it is not present in humans. iGb3 is synthesized by iGb3S, a member of the α1,3Gal/GalNAc transferase or Family 6 glycosyltransferases. Other family members include α1,3galactosyltransferase (α1,3 GT), and the B blood group transferase, which like iGb3S, transfer αGal. In contrast, the two other members of the family, A blood group transferase and Forssman synthetase, transfer αGalNAc. Members of Family 6 are the only known mammalian glycosyltransferases that transfer either αGal or αGalNAc in an α1,3 linkage to their respective acceptor molecules. Analysis of the human genome shows the genes for the α1,3GT, iGb3S, A/B blood group transferase, and the Forssman synthetase are present [19]. Recently, GT6m7, a new Family 6 member, was reported [19]; however, in humans, the gene for this glycosyltransferase contains a premature stop codon in the last exon. Indeed, mutation appears to be common in this family, with varying effects (see Table 1).

**Table 1:**
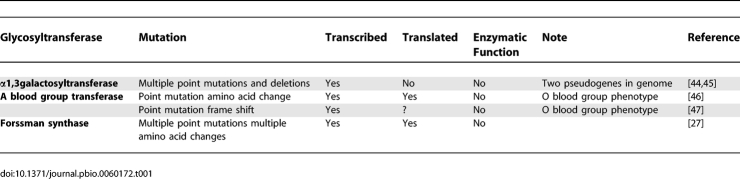
Characteristics of Human α1,3Gal/GalNAc (Family 6) Glycosyltransferases

In a similar fashion to ABO blood groups, in which natural antibodies are made to specificities that individuals lack, humans produce anti-αGal antibodies as a consequence of nonfunctional, or nontranslated enzymes. The evolutionary event that led to selection of the αGal-ve phenotype in humans is not clear, but selective pressure of the αGal+ve protozoan parasites has been postulated [20].

It is well known that the presence of the Galα(1,3)Gal xenoepitope, synthesized by α1,3galactosyltransferase (α1,3GT), causes hyperacute rejection of donor organs in pig-to-human xenotransplantation [21]. To avoid this problem, the α1,3GT gene has recently been deleted in pigs [22]. However, there is still low-level expression of Galα(1,3)Gal [23], presumably synthesized by iGb3S. Thus, organs from GT^−/−^ pigs transplanted into humans may still potentially be subject to rejection by human natural antibodies to Galα(1,3)Gal in the form of iGb3. The study from Zhou and colleagues [7] provided data suggesting that this is unlikely to pose an immediate problem, because they showed that human anti-Gal antibodies did not react with iGb3, presumably because it was a self-ligand that caused deletion of iGb3-reactive lymphocytes in humans. Thus, although there is clearly a significant level of controversy surrounding iGb3, the fact remains that there are compelling results both for and against a role for iGb3 in NKT cell development. For the sake of understanding the factors that regulate this process, as well as whether iGb3 poses an additional problem for xenotransplantion, further studies are required to resolve this issue.

**Figure 1:**
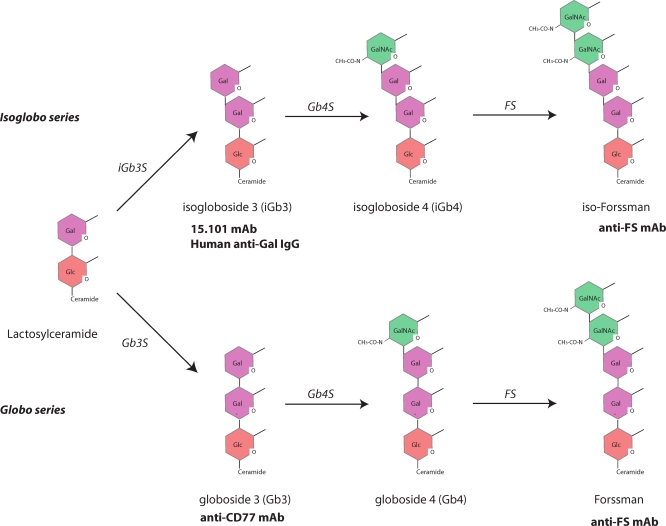
Lacosylceramide (Lac-Cer) Is the Starting Material for the Synthesis of Glycolipids via Two Distinct Pathways, the Isoglobo and Globo Series The major difference between the two pathways is that in the isoglobo series, iGb3S transfers an αGal in an α1,3 linkage, whereas in the globo series, Gb3S transfers an αGal in an α1,4 linkage. The relevant enzymes that catalyse the reactions to generate the different glycolipids are shown in italics. The antibodies that are used to detect these products are shown in bold.

We recently characterized the mouse iGb3S cDNA [24] that encodes the enzyme that synthesizes the Galα(1,3)Gal xenoepitope on iGb3 by catalysing the transfer of donor sugar from UDP-Gal in an α−1,3 linkage to its acceptor molecule Galβ(1,4)Glc-ceramide [25]. This reaction is the first step in the isoglobo-series pathway, which also results in the generation of iGb4 and isoForssman glycolipids (Figure 1).

Here, we have examined iGb3S expression and functional potential in human tissues. Moreover, to assess whether iGb3 might represent a xenoantigen that remains in α1,3GT knockout pigs, we investigated whether iGb3 glycolipid is recognized by natural human anti-Gal antibodies present in normal human serum, and we also determined whether cells expressing this glycolipid on the cell surface are readily targeted for complement-mediated lysis.

## Results

### Spliced iGb3S mRNA Is Not Detected in Any Human Tissue Analysed

**Figure 2:**
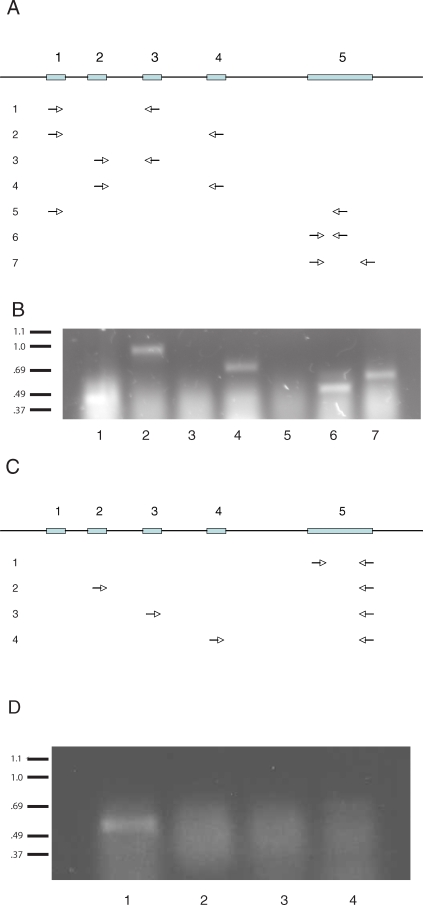
Spliced iGb3S mRNA Is Not Detected in Human Tissues (A) Schematic representation of the exon arrangement of human iGb3 synthase. Numbers (1–7) in the left-hand column denote the different primer combinations used in PCR analysis. Arrows define both primer direction and specific binding exons. (B) PCR products generated from dendritic cell cDNA template using the primer combinations described above. Numbers on the left are size markers (kilobases). (C) Schematic representation of the exon arrangement of human iGb3S. Numbers (1–4) in the left-hand column denote the different primer combinations used in PCR analysis. Arrows define both primer direction and specific binding exons. (D) PCR products generated from dendritic cell cDNA template using the primer combinations described above. Numbers on the left are size markers (kilobases). Using these and other primer combinations, these PCR experiments were repeated several times.

Several lines of evidence from our studies of the Galα(1,3)Gal epitope suggest that iGb3S is not expressed in humans. In contrast to both rat and mouse, in which the iGb3S gene is transcribed and the RNA processed [24,25], analysis of a human multiple tissue northern blot did not detect iGb3S mRNA (unpublished data)*.* Furthermore, anti-Gal monoclonal antibodies (mAbs) that detect both rat and mouse iGb3 on tissues and cell lines do not react with a range of both normal and malignant human tissues and cell lines [24] (and our unpublished data). To examine expression of iGb3S mRNA in greater detail, reverse-transcription PCR (RT-PCR) was used to analyse several human tissues. Oligonucleotide primers for these experiments (Table S1) were designed based on the exon arrangement of the human iGb3S gene, established by the analysis of Genbank DNA sequences. RNA from heart, kidney, spleen, lung, and thymus (the latter two tissues express iGb3S in both rat and mouse) generated a product of the correct size (∼550 bp) with forward and reverse primers within exon five (unpublished data). A product was also obtained from cDNA from human dendritic cells (Figure 2B, lane 6). Some products were confirmed as human iGb3S by direct sequencing (unpublished data). However, generation of products within an exon may be due to genomic DNA or heteronuclear RNA; therefore, amplification across exon boundaries is required to show the presence of mRNA. We have previously shown that iGb3S mRNA can be successfully used as a template for cross-exon RT-PCR from mouse RNA [24]. Despite exhaustive attempts (at least 50 times) to amplify human iGb3S using a combination of primers spanning all five exons in different tissues (Figure 2A), products were either not obtained from human template or the size of several of the PCR products corresponded to that expected from genomic DNA rather than spliced mRNA. The data shown are the amplification from dendritic cells (Figure 2B); however, similar results were also obtained from all tissues examined. Primers across exon 1 to 3, exons 1 to 4, and exons 2 to 4 yielded products expected from amplification of genomic DNA rather than spliced RNA (lanes 1, 2, and 4, respectively, Figure 2B and Table S2). The products observed in lanes 6 and 7 are within a single exon and could represent either genomic or mRNA products as there is no splicing over this region of the iGb3S gene. Despite using numerous primer combinations, including forward primers from exons 2, 3, or 4 with a reverse primer from exon 5 (Figure 2C), we were unable to detect any products of the correct size to suggest spliced iGb3S mRNA in any human tissue examined (Figure 2D), even when a high cycle number (up to 40) was used (unpublished data). As expected, products were not observed when template was omitted.

### Chimeric Human iGb3S Molecules Do Not Synthesize the Galα(1,3)Gal Xenoepitope

**Figure 3:**
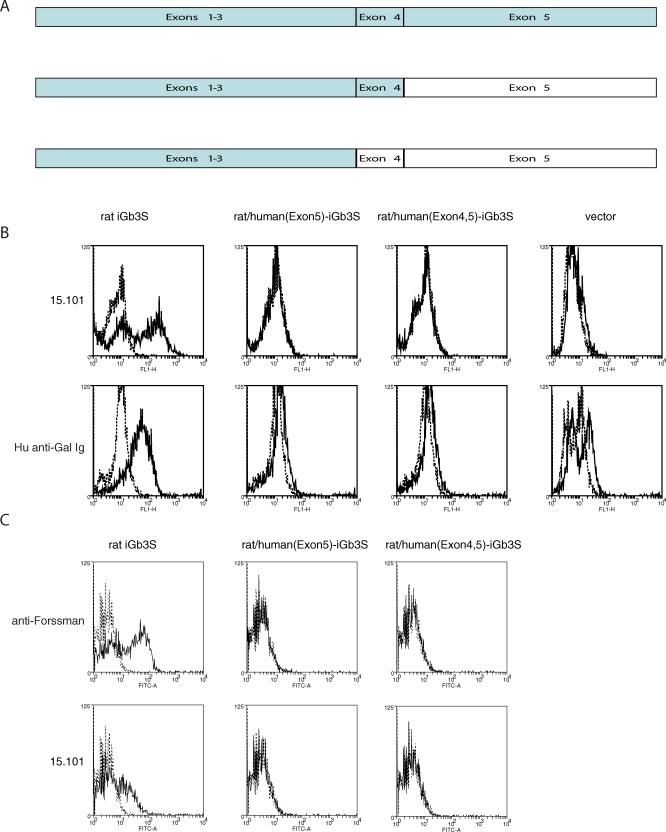
Chimeric Rat/Human-iGb3S Molecules Are Unable to Synthesize Galα(1,3)Gal or IsoForssman (A) Schematic diagram of rat iGb3S, chimeric rat/human(exon5)-iGb3S, and rat/human(exon4,5)-iGb3S. Rat exons are shaded grey; substituted human exons are unshaded. (B) Cell surface Galα(1,3)Gal expression after transfection with DNA encoding chimeric molecules (rat/human(exon5)-iGb3S and rat/human(exon4,5)-iGb3S) following staining with either the anti-Galα(1,3)Gal mAb 15.101 or natural human anti-Gal antibodies. The data are representative of five experiments. (C) IsoForssman and Galα(1,3)Gal expression following cotransfection with DNA encoding rat iGb3S or the chimeric molecules (rat/human(exon5)-iGb3S and rat/human(exon4,5)-iGb3S) and Forssman synthetase (FS). Cells were stained with either the anti-Forssman mAb or the Galα(1,3)Gal mAb 15.101. The data are representative of two experiments. Background staining with secondary antibody alone is shown by the dotted histograms in each panel.

From our experience, mouse iGb3S mRNA is expressed at low levels and is difficult to amplify. Therefore, our inability to detect human iGb3S mRNA was not conclusive evidence that it was absent. Transfection of CHOP cells with mouse iGb3S cDNA results in high level expression of its product Galα(1,3)Gal [24]. We used the same approach to determine whether humans express functional iGb3S. In vitro functional studies indicate that the catalytic domain of iGb3S (which represents 75% of the entire molecule) is encoded by two exons, a small exon (exon 4) and a larger one (exon 5) encoding the major part of the functional domain [26]. Soluble forms of the truncated catalytic domain of several members of the glycosyltransferase family have been shown to be enzymatically active. Using splice overlap extension PCR, we initially generated a chimeric molecule in which exon 5 of the functional rat iGb3S was substituted with that of the human iGb3S homolog (generated from human genomic DNA) (Figure 3A and Table S3). The other rat exons encode the cytoplasmic tail, transmembrane domain, and stalk that anchors the molecule in the lipid bilayer. This approach of exchanging catalytic domains to examine function has been successfully used with Forssman synthetase, another member of this glycosyltransferase family [27]. The ability of this chimeric rat/human(exon5)-iGb3S molecule to synthesize Galα(1,3)Gal was determined by analysis of transfected CHOP cells. As expected, cells transfected with DNA encoding rat iGb3S displayed strong cell surface expression of the Galα(1,3)Gal epitope on glycolipid as determined by binding of the monoclonal antibody 15.101 [28] and human anti-Gal immunoglobulin (Ig) purified from normal human serum (Figure 3B). The 15.101 mAb has been shown to bind preferentially to Galα(1,3)Gal on iGb3 lipid [28]. The chimeric molecule containing the majority of the catalytic domain of human iGb3S (rat/human(exon5)-iGb3S) was unable to synthesise the Galα(1,3)Gal epitope as staining was not observed with 15.101 or human anti-Gal Ig (Figure 3B). A second chimeric molecule comprising the entire human catalytic domain, exon 4 together with exon 5 (rat/human(exon4,5)-iGb3S), was also unable to synthesize Galα(1,3)Gal (Figure 3B). Data from several other mAbs and Bandeiraea simplicifolia IB4 lectin that bind the Galα(1,3)Gal epitope (Figure S1) support the conclusion that the human iGb3S catalytic domain is not functional. Detection of the FLAG epitope in both chimeric enzymes confirmed that the absence of Galα(1,3)Gal synthesis was not due to impaired translation or expression (Figure S2A). As glycosyltransferases are integral membrane proteins of the Golgi complex where oligosaccharides are synthesized, perinuclear staining (Golgi-like) confirmed correct trafficking of the chimeric enzymes (Figure S2B). Staining was not observed with cells transfected with vector alone.

### The Absence of iGb3 Staining Is Not Due to Antibody Inaccessibility or It Acting as a Precursor to Generate IsoForssman

To explore the unlikely possibility that iGb3 staining was not observed due to antibody inaccessibility, an alternative detection method was used. Synthesis of iGb3 is the initial step for the formation of the isoglobo-series glycolipid pathway, and iGb3 is the precursor to iGb4 and, ultimately, isoForssman [25] (see Figure 1). To examine whether iGb3 was synthesized, a complementation assay in CHOP cells, which lack both iGb3S and Forssman synthetase (FS), was used to determine whether coexpression of chimeric iGb3S with FS results in expression of isoForssman glycolipid. As expected, cells transfected with FS alone did not stain for isoForssman (unpublished data), whereas cells transfected with both rat iGb3S and FS were positive for isoForssman (Figure 3C). Expression of Galα(1,3)Gal was confirmed by 15.101 binding. Cells cotransfected with either of the chimeric molecules (rat/human (Exon5)-iGb3S or rat/human (Exon4,5)-iGb3S) and FS did not show any detectable isoForssman staining (Figure 3C). As expected, no Galα(1,3)Gal was observed following staining with 15.101. Thus, the human catalytic domain appears to be incapable of generating detectable iGb3 and does not initiate the downstream synthesis of the iGb4 structure required for FS to function*.*

### Pro 187 and Asn 252 in Human iGb3S Affect Enzymatic Function

**Figure 4:**
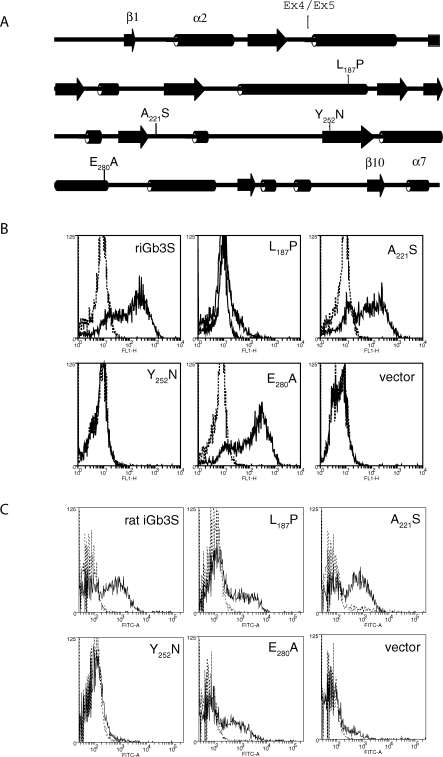
A Single Substitution in Rat iGb3S Completely Eliminates Galα(1,3)Gal Staining (A) Diagram, based on the crystal structure of rat iGb3S, showing the structure of exons 4 and 5 with the relative positions of the rat to human amino acid substitutions. The arrows represent beta sheets and the cylinders alpha helices. (B) Expression of Galα(1,3)Gal following staining with the anti-Galα(1,3)Gal mAb 15.101 of the rat to human point mutants (L_187_P, A_221_S, Y_252_N, and E_280_A). Background staining with secondary antibody alone is shown by the dotted histograms in each panel. The data are representative of five experiments. (C). IsoForssman expression following cotransfection of Forssman synthetase (FS) with DNA encoding the rat to human point mutants L_187_P, A_221_S, Y_252_N, and E_280_A. Cells were stained with the anti-Forssman mAb. The data are representative of three experiments. Background staining with secondary antibody alone is shown by the dotted histograms in each panel.

Using site-directed mutagenesis, we analysed which amino acid(s) contributed to the loss of function we observed in human iGb3S. Despite an overall similarity of approximately 72%, there are 77 differences within the catalytic domain of the functional rat iGb3S and nonfunctional human iGb3S, any of which, either alone or in combination, may be involved in the loss of function observed with human iGb3S. The targeted residues were selected by comparison of the aligned amino acid sequences of the iGb3S catalytic domains (exon 4 and 5 encoded) of species known to synthesize iGb3 (rat, mouse, and dog) with that of human. To identify more precise candidates, amino acids were excluded: (1) if the human amino acid was identical to either the mouse or dog, (2) the amino acid residue was different in all four species, or (3) the substitution was with an homologous amino acid. Four of these amino acids in rat exon 5 were selected in these initial studies and mutated to their human equivalent (Figure 4A). The single isolated substitution of rat Y_252_N resulted in the complete elimination of Galα(1,3)Gal staining (Figure 4B), showing that this asparagine in human iGb3S is sufficient to ablate enzymatic function. Rat L_187_P showed a significant reduction (typically 70%–95%) in Galα(1,3)Gal staining, whereas both the rat A_221_S and rat E_280_A substitutions showed strong Galα(1,3)Gal expression that was comparable with that observed following transfection with rat iGb3S (Figure 4B). As expected, a complementation assay with FS resulted in strong isoForssman staining with both rat A_221_S and rat E_280_A substitutions (Figure 4C). A similar high level of isoForssman staining was also observed with the rat L_187_P substitution, despite there being minimal Galα(1,3)Gal expression, thus demonstrating the sensitivity of this method. IsoForssman staining was not observed with cells cotransfected with rat Y_252_N (Figure 4C).

### Human iGb3S Is Inactive because of Multiple Mutations

**Figure 5:**
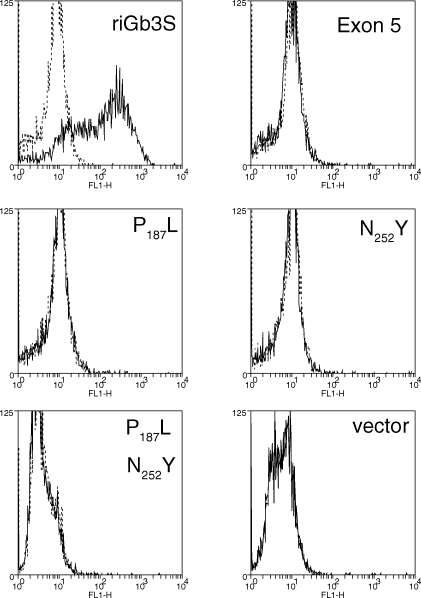
Human iGb3S Appears to Be Inactive because of Multiple Mutations Galα(1,3)Gal expression of the reverse human to rat single (P_187_L, N_252_Y) or combined point mutations (P_187_L+N_252_Y). Background staining with secondary antibody alone is shown by the dotted histograms in each panel. The data are representative of three experiments.

It is possible that the Y_252_N and L_187_P substitutions are not the only ones in humans that influence function. This was examined by reverse mutation of the nonfunctional chimeric rat/human(exon 5)-iGb3S to their rat equivalents with either point mutation alone (i.e., P_187_L or N_252_Y), or in combination (P_187_L+N_252_Y). A gain of function would suggest these are the primary residues involved in determining whether the transferase is functional. Staining with mAb 15.101 showed no Galα(1,3)Gal expression following transfection of CHOP cells with either the single or combined reverse-mutated chimeric cDNA molecules (Figure 5). The implications of these data are that human iGb3S must have multiple mutations that have resulted in its inactivation. Typical strong Galα(1,3)Gal expression was observed with cells transfected with rat iGb3S.

### Purified Human Anti-Gal Antibodies Bind iGb3 Lipid

**Figure 6:**
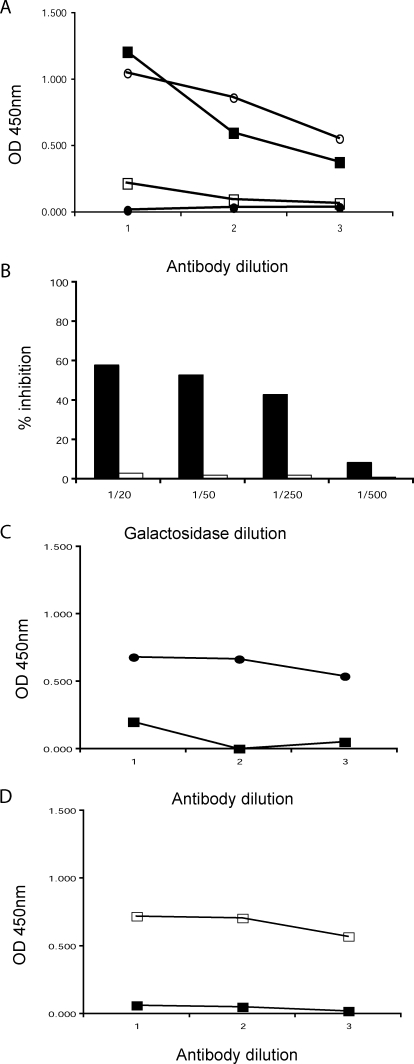
Purified Human Anti-Galα(1,3)Gal Antibodies Bind iGb3 Lipid (A) Doubling dilutions of natural purified human anti-Galα(1,3)Gal antibodies (0.49 mg/ml) (filled squares) (diluted 1/10) and anti-Galα(1,3)Gal mAb 15.101 supernatant (open circles) (diluted 1/2) binding to plate-bound iGb3 lipid. Background binding with anti-CD17 ascites (lactosylceramide) (diluted 1/40) and purified anti-CD77 (Gb3: 0.15 mg/ml) (diluted 1/50) is shown with open squares and closed circles, respectively. (B) Percent inhibition in binding of natural human anti-Galα(1,3)Gal antibodies to iGb3 lipid following α-galactosidase treatment at the dilutions shown. Unfilled columns represent the binding of anti-CD17 to plate-bound lactosylceramide after α-galactosidase treatment. (C and D) Binding of natural purified human anti-Galα(1,3)Gal antibodies (filled squares), anti-CD17 ascites (open squares), and purified anti-CD77 (closed circles) to either plate-bound Gb3 (C) or lactosylceramide (D). The data are representative of two experiments.

High-performance thin layer chromatography data from Zhou et al [7] suggested natural mixed human serum antibodies did not recognize iGb3, suggesting that iGb3-reactive B cells had been deleted from the human repertoire, further evidence that iGb3 lipid is present in humans. To test this ourselves, we used a lipid ELISA, and by this approach, demonstrated clear binding of both natural human anti-Galα(1,3)Gal antibodies and the mAb 15.101 to purified iGb3 lipid over several antibody dilutions (Figure 6A). Binding was not observed with either anti-CD17 (lactosylceramide) or anti-CD77 (Gb3) mAbs. Furthermore, as a specificity control, treatment of iGb3 lipid with α-galactosidase, which specifically removes the terminal α(1,3)Gal moiety, resulted in a significant inhibition (up to 60%) of binding by natural human anti-Galα(1,3)Gal antibodies (Figure 6B), yet had no effect on anti-CD17 binding to lactosylceramide (no αGal moiety) in a parallel assay. Antibody specificity was further demonstrated by the lack of binding of natural human anti-Galα(1,3)Gal antibodies to either Gb3 (Figure 6C) or lactosylceramide (Figure 6D). However, as expected specific binding of both anti-CD77 and anti-CD17 mAbs (used at the same dilutions as in Figure 6A) were observed (Figure 6C and 6D, respectively).

### Human Cells Expressing iGb3 Undergo Complement-Mediated Lysis

**Figure 7:**
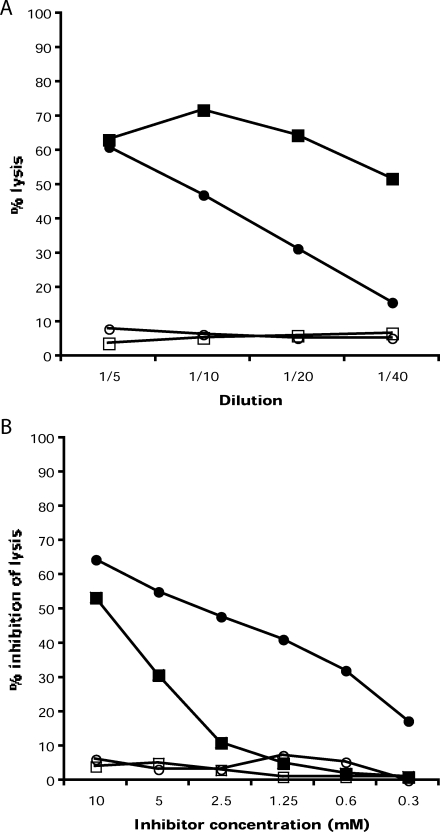
Complement-Mediated Lysis of Human Cells Expressing iGb3 (A) Percent lysis of human cells expressing iGb3 following addition of normal human serum (filled circles), purified anti-αGal IgG antibodies (filled squares) and NHS absorbed on Gal column (open circles) and nontransfected human cells following addition of NHS (open squares). Serum dilution is shown on the horizontal axis. Data are representative of three experiments. (B) Percent inhibition of lysis of human cells expressing iGb3 by preincubation of NHS with Galα(1,3)Gal disaccharide (closed circles) or lactose (open circles) or purified anti-αGal IgG antibodies with Galα(1,3)Gal disaccharide (closed squares) or lactose (open squares). Inhibitor dilution is shown on the horizontal axis. Data are representative of two experiments.

A key question that remains to be answered is, if human cells were to express iGb3, would they be susceptible to antibody-dependent complement-mediated lysis due to natural human anti-αGal antibodies present in normal human serum (NHS)? In contrast to nontransfected human cells (αGal^−ve^) that do not undergo lysis with NHS, human cells expressing iGb3 (αGal^+ve^) were lysed by NHS (in the presence of rabbit complement) in a dose-dependent manner (Figure 7A). Removal of anti-αGal antibodies from NHS by absorption with Galα(1,3)Gal coupled to glass beads abolished lysis to background levels (Figure 7A). However, lysis was not affected by NHS absorption with uncoupled glass beads (unpublished data). Furthermore, lysis of human cells expressing iGb3 was re-established when anti-αGal IgG antibodies were purified from NHS and used in the cytotoxicity assay (Figure 7A). In addition, this activity could be removed by absorption with Galα(1,3)Gal coupled to glass beads (unpublished data). Inhibition experiments verified that Galα(1,3)Gal is the epitope that the antibodies recognise, as a significant dose-dependent reduction in lysis was observed by preincubation of both NHS and anti-αGal IgG antibodies with Galα(1,3)Gal disaccharide (Figure 7B). No inhibition was observed when lactose (Galβ(1,4)Glc) was used (Figure 7B).

## Discussion

Glycolipids represent one of the last molecular frontiers in immunological recognition. Whereas glycolipids are known to be synthesized in the Golgi and are typically expressed on the cell surface, the exact transport pathway(s) for newly synthesized glycolipids is not well defined. However, it is assumed to be similar to glycoproteins and involve vesicular flow from the endoplasmic reticulum through the Golgi complex to the plasma membrane. Glycolipids, particularly exogenous glycolipids, can localize to lysosomal compartments via endocytosis. Similarly, our knowledge of how glycolipids control immune responses and the context in which they are presented by CD1d and recognized by NKT cells is also still very limited. Since the glycolipid, α-GalCer, was originally shown to potently stimulate NKT cells in a CD1d-dependent manner, there has been an enormous effort to identify other ligands. Several classes of natural CD1d-binding ligands for NKT cells have been identified, including microbial-derived α-linked glycosphingolipids from the nonpathogenic *Sphingomonas* bacteria and phosphatidylinositol mannoside from *Mycobacteria* (reviewed in [29]). Recently, a diacylglycerol glycolipid from Borrelia burgdorferi, a human pathogen responsible for Lyme disease, was shown to directly stimulate both human and mouse NKT cells [30]. Although these ligands are all candidates for NKT cell recognition of non-self, none of these are present in normal mammalian cells.

The main candidate self glycolipid-antigen is iGb3. The original collective data, primarily based on the use of β-hexosaminidase-B–deficient mice that are incapable of degrading iGb4 into iGb3 in lysosomes, supported the claim that iGb3 lipid was a principle endogenous ligand for Vα14 NKT cells in mice and, albeit indirectly, in humans [7,12,31]. The interpretation of data using β-hexosaminidase-B–deficient mice was contested by Gadola et al. [14], where it was argued that these mice have a generalised lysosomal storage disease that indirectly impaired CD1d loading in lysosomes. Their interpretation was that it was the accumulation of glycolipids in lysosomes, rather than the lack of iGb3, that abrogated NKT cell development. Some of the data in this paper [14] simply conflicted with that of the earlier study of β-hexosaminidase-B–deficient mice [7], making it difficult to determine which interpretation was correct [14]. Similar suggestions were raised in an independent study of mutations leading to lysosomal storage diseases [18]. Recently, Porubsky et al. and Speak et al. [15,16] failed to detect iGb3 in mouse and human thymus using a biochemical approach. Furthermore, Porubsky et al. [15] reported normal development and function of invariant NKT (iNKT) cells in iGb3S^−/−^ mice. Although the lack of biochemical evidence for iGb3 in thymus might simply be an issue of insufficient sensitivity, the results from the iGb3S^−/−^ mice more strongly challenge the significance of iGb3 in mouse NKT cell development. There is no easy interpretation that incorporates and integrates the findings from the studies for, and against, a role for iGb3 in mouse NKT cell development. In our opinion, this represents one of the most important and controversial issues in the NKT cell field that requires additional input from independent research groups.

In humans, synthetically derived iGb3 can stimulate human NKT cells to proliferate and produce cytokines [7,8,32] and recognition of human dendritic cell self-antigen can be blocked by IB4 lectin [7]. However, direct biochemical evidence to show that human iGb3 is an endogenous NKT cell ligand has been lacking. Although iGb3 was not detected in human thymus or human dendritic cells using a high-performance liquid chromatography (HPLC) assay, this assay had a detection limit of 1% iGb3 to 99% Gb3, which does not exclude the presence of iGb3 at low but still biologically significant levels [16]. Indeed, during review of this manuscript, two publications from Li et al., claimed to be able to discriminate iGb3 from Gb3 (in artificial mixtures and from rat cells) and identified iGb4 from human paediatric thymi, using electrospray ionisation-ion trap mass spectrometry [33,34]. Although these analyses are at odds with our own, they have yet to conclusively demonstrate immunologically significant levels of iGb3 in human tissue. Specifically, the formal possibility remains that the minor MS^n^ mass spectral signature for iGb4 detected in these studies is derived from related tetraglycosylceramides, as acknowledged by these investigators. Alternatively, the very low levels of iGb4 detected in these analyses may be derived from dietary sources and distributed throughout the body via lipoprotein particles.

The presence or absence of iGb3 in humans has potential major implications for xenotransplantation. If humans express iGb3S, iGb3 lipid present on transplanted pig tissues will not be “seen” as foreign and therefore would not represent a drawback for xenotransplantation. However, as humans do not express functional iGb3S (reported herein), then the presence of lipid-linked Galα(1,3)Gal in pigs, synthesized by iGb3S, may pose a serious risk to successful xenotransplantation, even when using α1,3GT knockout pigs as donors (which were specifically generated to eliminate Galα(1,3)Gal epitopes for xenotransplantation purposes). What are the implications of this in a transplant setting? Currently, we know that expression of iGb3 does not mediate hyperacute rejection of pig tissues transplanted into baboons [35,36]. However, human serum has at least a 4-fold higher level of natural anti-Galα(1,3)Gal antibodies (∼1% of human IgG) than other primates [37]**,** so this may not directly represent the human situation. Furthermore, iGb3 expression in pigs may have more serious consequences in the later phases of graft rejection. Firstly, changes in the affinity/avidity of the elicited antibodies may cause tissue damage by complement fixation. It is clear that the level of anti-Galα(1,3)Gal antibody is critical for the speed of rejection in experimental models [38]. Alternatively, elicited anti-Galα(1,3)Gal antibodies may contribute to the acute vascular rejection observed when hyperacute rejection is eliminated, such as in knockout pig-to-primate transplants, by activation of endothelial cells via cross-linking of the lipid itself. Pathological features similar to acute vascular rejection are seen in humans when the Gb3 lipid (closely related to iGb3) is cross-linked by bacterial toxins [39]. Secondly, because iGb3 activates human NKT cells [7,32] (in which synthetic, purified. and enzymatically derived iGb3 were all tested), consequently the expression of iGb3 on pig cells could lead to NKT cell activation resulting in destruction of the xenograft. Furthermore, our data clearly show that the anti-Gal antibodies in NHS can lyse iGb3 expressing cells (Figure 7) and therefore any remaining iGb3 on pig cells may be a target for antibody-mediated destruction. Whereas it is clear that the use of heavy immunosuppression can control the later phases of xenograft rejection, the major advantage of xenotransplantation over allotransplantation is the ability to genetically modify the donor. The ultimate goal is to engineer a donor pig such that minimal, or indeed no immunosuppression is required for long-term graft survival. It is likely that genetic modification of pigs may be required to eliminate any effects of iGb3. Only at that stage will other obstacles be revealed. Thus, in formally demonstrating the lack of functional iGb3S in humans, this study alerts transplantation immunologists to a previously unrecognised risk associated with expression of iGb3 glycolipid on α1,3GT knockout pig tissues. Expression of this glycolipid could act as a secondary source of Galα(1,3)Gal xeno-antigen capable of binding natural human anti-Gal antibodies present in normal human serum and marking these cells for destruction by complement mediated lysis.

In a perspectives article, Godfrey, Pellicci, and Smyth [40] asked whether the search for the elusive NKT cell antigen is over. In mice, in view of several recent publications [14–16], the possible existence of NKT cell-selecting ligands other than iGb3 remains an important consideration [17]. It had generally been assumed that experimental data obtained from mice would be directly relevant to humans, and Zhou et al. [7] provided indirect evidence that iGb3 is also a self-ligand for human NKT cells. However, our new data demonstrate that there appears to be critical differences between the two systems, and suggests that we are a long way from calling off the search for NKT cell-selecting antigens in humans. This remains one of the most important objectives in the field, and will ultimately lead to a better understanding of the factors that regulate NKT cell development and function in health, and in developing novel therapies for the treatment of disease.

## Materials and Methods

### Genomic database searching.

The human genomic iGb3S sequence was obtained from the National Center for Biotechnology Information Web site (http://www.ncbi.nlm.nih.gov) and searching the human genomic database. The nucleotide sequence of the gene *A3GALT2* (accession number NT 032977) was used, with the exon/intron boundaries for the human iGb3S gene as listed with the sequence.

### PCR.

We were unable to clone human iGb3S from total RNA from adult human tissues (heart, lung, kidney, spleen, and thymus) (Stratagene) or cDNA from dendritic cells using the TITANIUM One-Step RT-PCR Kit (Clontech) with a series of degenerate primers (Tables S1 and S2). The chimeric rat/human molecules included the exchange of rat exon 5 (rat/human(exon5)-iGb3S) and rat exons 4 and 5 (rat/human(exon4,5)-iGb3S) with the equivalent human exon(s). The rat/human chimeras were generated using splice overlap extension PCR. The specific primer combinations used are shown in Table S3. The single amino acid substitutions in rat iGb3S (L_187_P, A_221_S, Y_252_N, and E_280_A) and the reverse mutations in rat/human(exon5)-iGb3S (P_187_L, N_252_Y, and the combined P_187_L+N_252_Y) were introduced using the QuikChange site-directed mutagenesis kit (Stratagene) (Table S4). Sequence fidelity, orientation of the insert, and presence of the desired mutation(s) were confirmed by DNA sequencing (Big Dye 3.1; PE-Applied Biosystems).

###  Cell culture, transfection, and antibodies.

CHOP cells (Chinese Hamster Ovary cells transformed with Polyoma Large T antigen) [41] and E293 cells (human kidney fibroblasts) were cultured in DMEM (CSL) supplemented with 10% FCS overnight at 37 °C. Transfections were with LipofectAMINE Plus (Life Technologies) as recommended by the manufacturer. Cells were examined after 48 h for either cell surface or intracellular expression of Galα(1,3)Gal using purified natural human anti-Gal antibodies (0.49 mg/ml), and the anti-Galα(1,3)Gal mAbs 15.101, 22.121, 24.7, 25.2, and 8.17 (supernatants) [24,42] and Bandeiraea simplicifolia IB4 lectin. Expression of the FLAG epitope was revealed by staining with the anti FLAG M2 mAb (Sigma). Expression of IsoForssman glycolipid was revealed using an anti-Forssman mAb, FOM-1 (BMA Biomedicals). Expression of lactosylceramide and Gb3 were revealed with anti-CD17 (ascites) and purified anti-CD77 (0.15 mg/ml) mAbs, respectively (Pharmingen). Antibodies were detected with FITC-labelled sheep anti-mouse or human IgG (Dako) or HRP conjugate Sheep anti-human Ig (Silenus) and rabbit anti-mouse Ig (Dako), and analysed either by fluorescence microscopy, flow cytometry (Becton Dickinson FACS Canto II), or lipid ELISA.

### Lipid ELISA and enzyme digestion.

Porcine lactosylceramide (Calbiochem) and iGb3 (Alexis Biochemicals) were dissolved in methanol at 1 mg/ml and stored at −20 °C. The ELISA was performed in 96-well Maxisorb plates (Nunc). Lipids were diluted in *n*-hexane and used at 500-ng/well, incubated for 1 h in a fume hood to dry; plates were then blocked with 3% BSA/PBS for 2 h and washed ×1 with PBS. Primary antibodies, diluted in blocking buffer, were added and incubated for 1 h. After washing ×5 with PBS, secondary antibodies, diluted in blocking buffer, were added and incubated for 1 h before washing ×8 with PBS. All incubations were carried out at room temperature (RT) on a rocking platform. TMB peroxidase substrate (KPL) was used to develop the plate. Colour development was stopped with 0.18 M H_2_SO_4_ and quantitated at an optical density at 405 nm (OD_405nm_) on an ELISA plate reader. For the enzyme digestion, α-galactosidase (Sigma-Aldrich) was diluted in 0.1 M citrate/phosphate buffer (pH 6) and incubated with the lipids overnight at RT, after which an ELISA was performed as described above.

### Complement-mediated lysis.

Human E293 cells expressing rat iGb3 [28] were tested for lysis with rabbit complement and normal human sera (NHS, pooled from ten healthy individuals and heat inactivated) or purified human anti-Gal IgG antibodies (prepared by fractionation of the NHS pool on a Protein G Sepharose column (Pharmacia) followed by affinity chromatography on Galα(1,3)Gal-coupled macroporous glass beads (Syntesome) as described previously [43]. In brief, 50 μl of antibody at doubling dilutions were added to 2.5 × 10^5^ cells per well in round-bottomed 96-well plates (Greiner), resuspended, and incubated on ice for 30 min. After two washes, 50 μl of rabbit complement, at an appropriate dilution, was added to the cell pellet, resuspended, and incubated at 37 °C for 30 min (NHS) or 60 min (purified anti-Gal IgG). Cells were pelleted and resuspended in 400 μl of DMEM/0.5% BSA containing 1 μg/ml propidium iodide (PI; Sigma) and analysed by flow cytometry. Percentage lysis (cytotoxicity) was determined by analysis of 10,000 cells.

The importance of anti-Gal antibodies for lysis was determined by serum absorption and carbohydrate inhibition: (1) Absorption; 200 μl of NHS or human anti-Gal IgG was added to an equal volume of Galα(1,3)Gal-coupled macroporous glass beads or non-coupled beads (control) at 4 °C for 30 min; the beads were removed by centrifugation and the absorption step repeated with another aliquot of beads. (2) Carbohydrate inhibition; 25 μl of 20 mM Galα(1,3)Gal disaccharide or lactose (control) was serially diluted and mixed with an equal volume of NHS or human anti-Gal IgG at an appropriate dilution (two dilutions less than the 50% titre of the antibody) and incubated at 4 °C for 16 h. After both of these treatments, the sera were analysed for complement-mediated lysis.

## Supporting Information

Figure S1 Cell Surface Staining of Transfected Cells Using Monoclonal Antibodies Shows Galα(1,3)Gal expression on cells expressing rat iGb3S, rat/human(Exon5)-iGb3S, or rat/human(Exon4,5)-iGb3S following staining with a panel of the anti-Galα(1,3)Gal mAbs 22.121, 24.7, 25.2, or 8.17 and the IB4 lectin. Background staining with secondary antibody alone is shown by the dotted histograms in each of the 16 panels. The data are representative of two experiments.
(69 KB PDF)

Figure S2 Chimeric Protein Expression after Transfection (A) shows a western blot showing anti-FLAG staining of lysates from mock-transfected CHOP cells (lane 1), cells transfected with rat/human(Exon4,5)-iGb3S (lane 2), or rat/human(Exon5)-iGb3S (lane 3). The arrow represents the expected size of the chimeric proteins (40 kD) observed in lanes 2 and 3. The darker, lower band observed in all wells is nonspecific.
(B) Fluorescence microscopy showing perinuclear (Golgi-like) staining of rat iGb3S (panel 1), rat/human(Exon4,5)-iGb3S (panel 2), and rat/human(Exon5)-iGb3S (panel 3).
(128 KB PDF)

Table S1 Primers Used in PCR Analysis of Human Tissues and in the Generation of Chimeric Rat/Human iGb3S (58 KB DOC)

Table S2 PCR Primer Combinations Used to Analyse Dendritic Cell (DC) cDNA The first primer in each of the combinations is the forward primer. Predicted genomic sizes were calculated using the original Genbank data file NT004511.16.
(31 KB DOC)

Table S3 Primer Combinations Used in Generating Chimeric Rat/Human-iGb3S-Flag cDNAs (29 KB DOC)

Table S4 Primers for Generating Specific Amino Acid Changes in Rat iGb3S and Chimeric Rat/Human(exon5)-iGb3S (39 KB DOC)

## Abbreviations

FS: Forssman synthetase
IB4: isolectin B4
Ig: immunoglobulin
iGb3: isogloboside 3
iGb3S: isogloboside 3 synthase
mAb: monoclonal antibody
NHS: normal human serum
NKT: natural killer T
α1,3GT: α1,3galactosyltransferase
