# Supplementary material for: Humans Lack iGb3 Due to the Absence of Functional iGb3-Synthase: Implications for NKT Cell Development and Transplantation
Source: PLoS Biol. 2008 Jul 15;6(7):e172. doi: 10.1371/journal.pbio.0060172 (PMC2459210; doi:10.1371/journal.pbio.0060172)
Supplement: Supplementary file 1 [file pbio.0060172.sg001.pdf]

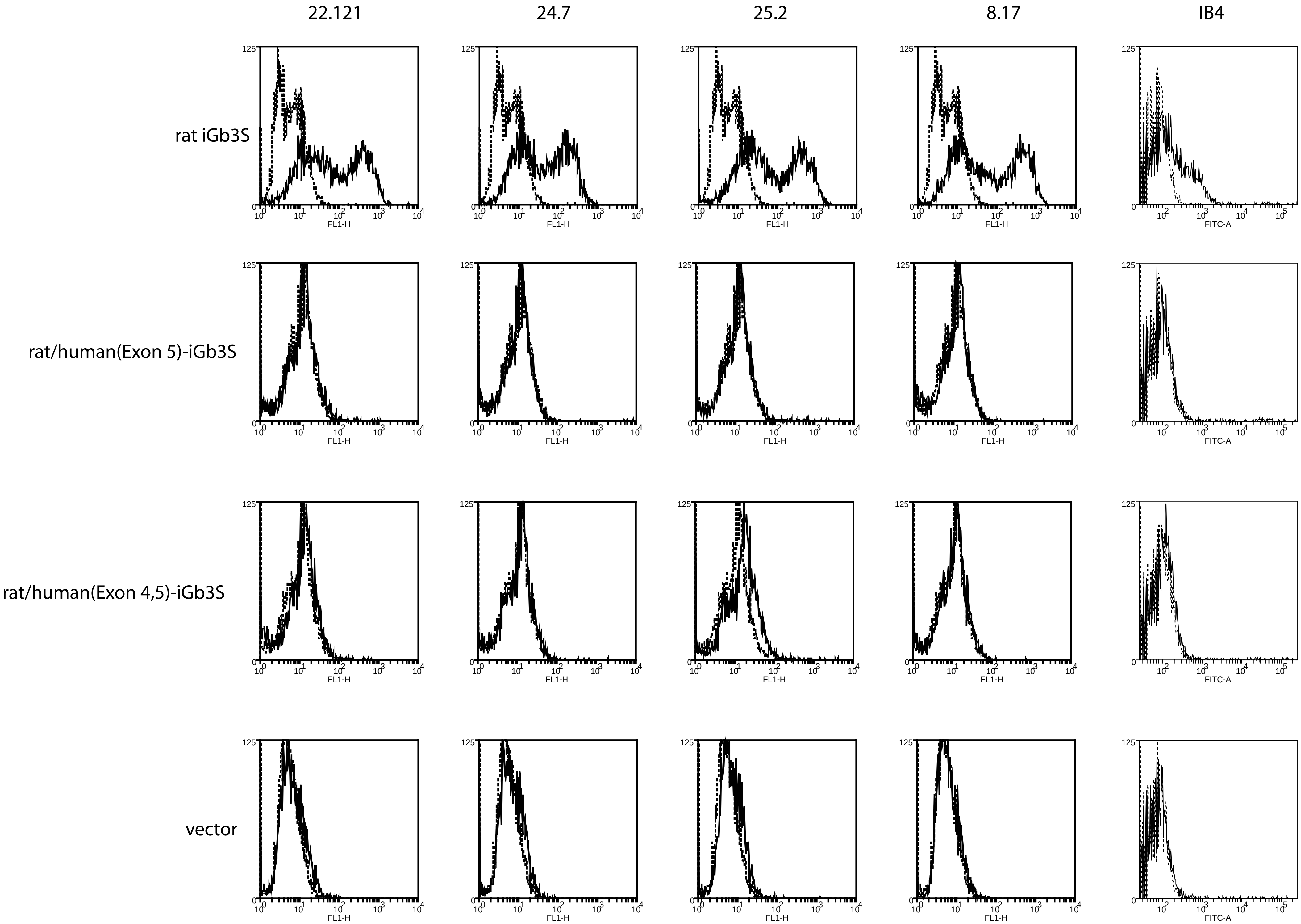
22.121
24.7
25.2
8.17
IB4
rat iGb3S
rat/human(Exon 5)-iGb3S
rat/human(Exon 4,5)-iGb3S
vector
FL1-H
FITC-A
