# Supplementary material for: Humans Lack iGb3 Due to the Absence of Functional iGb3-Synthase: Implications for NKT Cell Development and Transplantation
Source: PLoS Biol. 2008 Jul 15;6(7):e172. doi: 10.1371/journal.pbio.0060172 (PMC2459210; doi:10.1371/journal.pbio.0060172)

FigS2

A

1 2 3

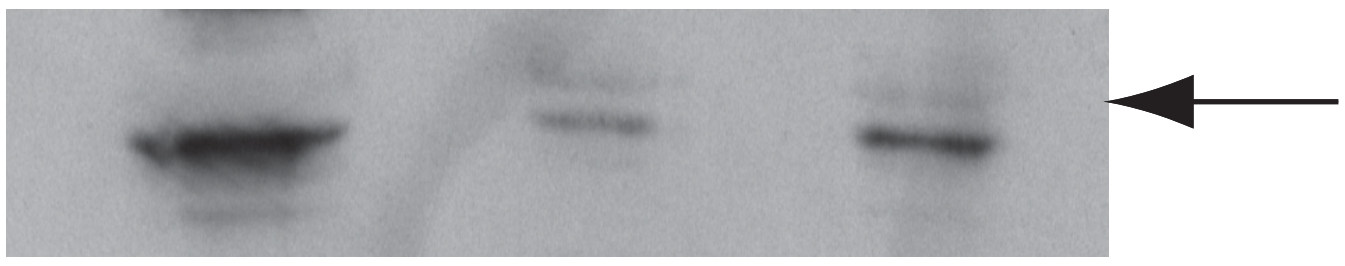

B

1 2 3

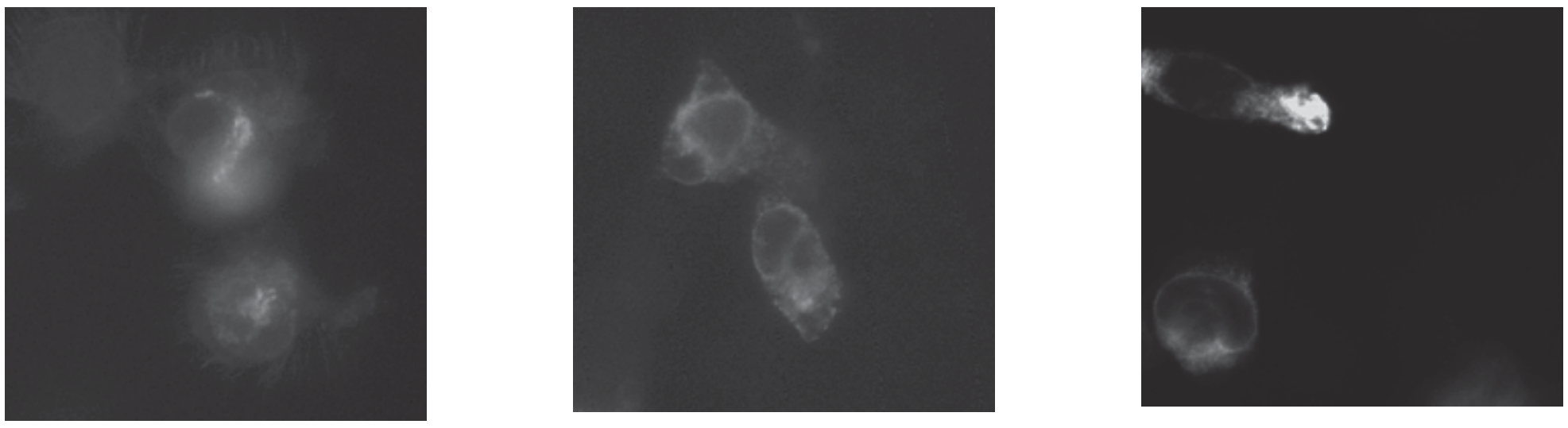

Supplement: Supplementary file 2 [file pbio.0060172.sg002.pdf]
