# Supplementary material for: Humans Lack iGb3 Due to the Absence of Functional iGb3-Synthase: Implications for NKT Cell Development and Transplantation
Source: PLoS Biol. 2008 Jul 15;6(7):e172. doi: 10.1371/journal.pbio.0060172 (PMC2459210; doi:10.1371/journal.pbio.0060172)
Supplement: Supplementary file 3 [file pbio.0060172.st001.doc]

| Primer | Sequence (5’-3’) | **Direction** | **Binding Exon** |
| --- | --- | --- | --- |
| MO886 | CCTGGAGAAGTACCTGGAGCGCTTC | forward | 5 |
| MO887 | CTTGTGCAGCCAGAAGAACTTGTTG | reverse | 5 |
| MO903 | CTGTTTCTGTATGGCCTCCCTAAATTC | forward | 2 |
| MO904 | CATCTGGAAGCCCTCATCCCCATG | forward | 3 |
| MO905 | CGGCCTGAAGTTCTGACCTGTACC | forward | 4 |
| MO906 | CTAGTTCCGCAGCAGCCGGTACCC | reverse | 5 |
| MO908 | CGTCTCCAGGAAGCGCTCCAGGTACTTCTCCAGGTACCTGCCTACCGC | reverse | overlapping |
| MO909 | GCGGTAGGCAGGTACCTGGAGAAGTACCTGGAGCGCTTCCTGGAGACG | forward | overlapping |
| MO910 | GCCTAGTTCCGCAGCAGCCGGTACCC | reverse | 5 |
| MO911 | CCATGGCTCTGGAGGGACTCAGGGCC | forward | 1 |
| MO914 | ATGCCCCTGCACAAGAAACTGGTAAC | forward | 1 |
| MO915 | CTGTTTCTGTATGGCCTCCCTAAATTC | forward | 2 |
| MO916 | CATGGGGATGAGGGCTTCCAGATGCC | reverse | 3 |
| MO917 | GCAAAGATAGTCAGCCCAATGGTGAGG | reverse | 4 |
| MO918 | GGCCACATCTGGGTCGAAAGAGCCATCCCAAATAA-TGGGAGCCCCCCAGGGGGTACAGGTCAGAACTTCA-GGCCGGGCCCAGTGACGCAGCACTCCGGT | reverse | overlapping |
| MO919 | TCTTTCGACCCAGATGTGGCCAAGCAAGAGGCTAG-ACAGCAGAACCTCACCATTGGGCTGACTATCTTTGC-TGTAGGCAGGTACCTGGAGAAGTACCTG | forward | overlapping |
| MO922 | GGCTACTTGTCATCGTCGTCCTTGTAGTCGTTCCGCAGCAGCCGGTACCC | reverse | 5 |
