# Supplementary material for: Humans Lack iGb3 Due to the Absence of Functional iGb3-Synthase: Implications for NKT Cell Development and Transplantation
Source: PLoS Biol. 2008 Jul 15;6(7):e172. doi: 10.1371/journal.pbio.0060172 (PMC2459210; doi:10.1371/journal.pbio.0060172)
Supplement: Supplementary file 4 [file pbio.0060172.st002.doc]

| Lane | Primer Combination | Genomic Predicted size (bp) | cDNA Predicted size (bp) | Observed (bp) |
| --- | --- | --- | --- | --- |
| 1 | MO914+MO916 | 477 | 117 | ~490 |
| 2 | MO914+MO917 | 982 | 314 | ~1000 |
| 3 | MO903+MO916 | 243 | 53 | - |
| 4 | MO903+MO917 | 775 | 247 | ~750 |
| 5 | MO914+MO906 | 6278 | 1011 | - |
| 6 | MO886+MO887 | 568 | 568 | ~550 |
| 7 | MO886+MO906 | 685 | 685 | ~690 |
