# Supplementary material for: Humans Lack iGb3 Due to the Absence of Functional iGb3-Synthase: Implications for NKT Cell Development and Transplantation
Source: PLoS Biol. 2008 Jul 15;6(7):e172. doi: 10.1371/journal.pbio.0060172 (PMC2459210; doi:10.1371/journal.pbio.0060172)
Supplement: Supplementary file 5 [file pbio.0060172.st003.doc]

| Construct | Primers | Template |
| --- | --- | --- |
| Rat/human(exon5)iGb3S-Flag | 886+906 | human genomic DNA |
|  | 909+910 | product of 886+906 |
|  | 911+908 | ratiGb3 cDNA |
|  | 911+922 | products: (909/910)+(911/908) |
| Rat/human(exon4,5)iGb3S-Flag | 911+918 | ratiGb3 cDNA |
|  | 919+922 | product of 909+910 |
|  | 911+922 | products: (911/918)+(919/922) |
