# Supplementary material for: Humans Lack iGb3 Due to the Absence of Functional iGb3-Synthase: Implications for NKT Cell Development and Transplantation
Source: PLoS Biol. 2008 Jul 15;6(7):e172. doi: 10.1371/journal.pbio.0060172 (PMC2459210; doi:10.1371/journal.pbio.0060172)
Supplement: Supplementary file 6 [file pbio.0060172.st004.doc]

| Substitution (from–to) | Primer | Sequence (5’ – 3’) | Direction |
| --- | --- | --- | --- |
| L - P | MO941 | CTGGGAGGGCAGCCGGGCCGAGAAGCT | forward |
|  | MO942 | AGCTTCTCGGCCCGGCTGCCCTCCCAG | reverse |
| A - S | MO945 | GCACAGCTGCACTCCTGGCACTTCCGC | forward |
|  | MO946 | GCGGAAGTGCCAGGAGTGCAGCTGTGC | reverse |
| Y - N | MO949 | GGCGATTTCTACAACCACGCCGCGGTG | forward |
|  | MO950 | CACCGCGGCGTGGTTGTAGAAATCGCC | reverse |
| E - A | MO951 | CAGCTGGACCTGGCGCATGGCATTGAG | forward |
|  | MO952 | CTCAATGCCATGCGCACGGTCCAGCTG | reverse |
| P - L | MO993 | CTGGGCGGGCTGCTGGGCCGCGAGGCG | forward |
|  | MO994 | CGCCTCGCGGCCCAGCAGCCCGCCCAG | reverse |
| N - Y | MO995 | GGCGACTTCTATTACCACGCGGCGGTG | forward |
|  | MO996 | CACCGCCGCGTGGTAATAGAAGTCGCC | reverse |
